# Influence of Pre-Pregnancy Obesity on Carbohydrate and Lipid Metabolism with Selected Adipokines in the Maternal and Fetal Compartment

**DOI:** 10.3390/nu15092130

**Published:** 2023-04-28

**Authors:** Andrzej Miturski, Tomasz Gęca, Aleksandra Stupak, Wojciech Kwaśniewski, Anna Semczuk-Sikora

**Affiliations:** 1 Department of Gynaecology, 1st Clinical Military Hospital in Lublin, Al. Racławickie 23, 20-049 Lublin, Poland; 2 Chair and Department of Obstetrics and Pathology of Pregnancy, Medical University of Lublin, Staszica 16 Street, 20-081 Lublin, Poland; 3 Department of Gynecologic Oncology and Gynecology, Medical University of Lublin, Staszica 16 Street, 20-081 Lublin, Poland

**Keywords:** carbohydrate metabolism, lipid metabolism, obesity, macrosomia, adipokines, pregnancy

## Abstract

A higher body mass index (BMI) before pregnancy is associated with an increased risk of maternal and perinatal complications. This study aimed to analyze selected parameters of carbohydrate and lipid metabolism, including adipokines, in obese pre-pregnant women, and their influence on the birth weight of newborns. Materials and Methods: The study group (O) consisted of 34 pregnant women with higher BMI (obese) before pregnancy. The control group (C) was 27 pregnant women with target BMI and physiological pregnancy. The BMI index: body weight [kg]/(height [m]^2^ was assessed on the first obstetrical visit. The research material was the serum of pregnant women collected in the third trimester of pregnancy and umbilical cord blood collected immediately after delivery. Selected parameters of carbohydrate and lipid metabolism and adipokines were determined. Results: There were no statistically significant differences between the study group and the control group concerning the concentrations of insulin, glucose, VLDL, adiponectin, TNF-α, HOMA-IR, as well as LDH and cholesterol in maternal blood serum and umbilical cord blood serum. Total cholesterol and HDL in both maternal blood serum and umbilical cord blood were statistically significantly lower than those in the control group. The concentration of triglycerides (TG) and resistin in the blood serum of obese mothers were higher than those in the control group (*p* < 0.05). However, no statistically significant differences were found between the two groups regarding the concentrations of TG and resistin in the umbilical cord blood. The concentration of LDL cholesterol in the umbilical blood serum in the obese group was statistically significantly lower than that in the control group. The concentration of leptin in maternal blood serum and umbilical cord blood serum in the study group was statistically significantly higher than that in the control group. Conclusions: Pregestational obesity does not substantially affect the basic parameters of carbohydrate metabolism in pregnant women, but it disturbs the lipid profile, which is manifested by a significant increase in triglycerides and a decrease in the level of HDL cholesterol in the serum. Preexisting obesity increases the concentration of leptin and resistin in the serum of pregnant women, which may be caused by the increased volume of adipose tissue. The concentrations of leptin and resistin in the blood of pregnant women correlate positively, and the concentrations of adiponectin and TNF-α negatively correlate with pre-pregnancy BMI values. There is a positive correlation between the concentration of leptin in the serum of umbilical cord blood and the birth weight of the newborn, which suggests that this parameter contributes to the pathomechanism of macrosomia.

## 1. Introduction

### 1.1. Obesity as a Clinical Problem

Obesity in women is an important element that determines the health of a future mother and her newborn baby [1]. A commonly used definition of obesity was proposed by the WHO in 1997 [2]. It is described as a condition characterized by an increase in body weight through hypertrophy and/or hyperplasia of adipose tissue, above 25% of the proper body weight in men and over 30% in women, posing a health risk [3]. One of the most frequently used weight indicators is Quetelet II, more commonly known as the Body Mass Index (BMI). It is calculated according to the following formula: body weight [kg]/(height [m^2^]). Overweight is defined as a BMI of 25 to 29.9, and obesity is defined as a BMI of 30 kg/m^2^ or higher [4]. The pathogenesis of obesity is complex, where genetic, environmental, social, cultural, metabolic, and endocrine factors play equal roles [5]. It has been proven that the excessive bodyweight of one of the parents increases the risk of obesity in a child by four–five times, and obesity of both of parents can even increase the risk by thirteen times [6].

The risk of obstetric complications, especially in the perinatal period, increases with the mother’s BMI by increasing the number of cesarean sections and surgical deliveries, gestational diabetes, and large for gestational age neonates [7,8]. Maternal obesity may also cause later health problems for the child, including the risk of obesity in the preschool age. The literature suggests that not only obesity itself, but also excessive weight gain especially in the third trimester of pregnancy, increases the frequency of these complications—particularly in the cases of overweight and obese women diagnosed before pregnancy [9]. Su et al. revealed that macrosomic newborns are born almost exclusively to obese mothers or mothers with excessive weight gain during pregnancy [10]. A similar tendency was also observed in the conducted studies, confirming the occurrence of macrosomia in 35% of newborns in pre-pregnancy obese women and only in 7.4% of children of mothers with lower body weight.

Obesity causes regulatory disturbances in many metabolic pathways including carbohydrate metabolism, and especially insulin. Obese women have higher serum levels of this hormone compared with those with lower body weight [11,12]. The literature indicates that it is mainly due to a chronic subclinical inflammatory process occurring systemically, especially in insulin-sensitive tissues, including skeletal muscle and adipose tissue [13]. However, it should not be forgotten that the increase in insulin concentration in a pregnant woman and the intensification of insulin resistance are a physiological change during pregnancy, ensuring the proper delivery of nutrients to the fetus [11,12].

The theory of “intrauterine programming” assumes that some features, such as the intrauterine environment or genetic factors, can affect the ability of the maternal-placental transport of substances responsible for the growth and development of the fetus [14]. Pre-pregnant obesity also increases the risk of developing diseases later in life, especially type 2 diabetes [7,15,16]. It has been found that newborns of obese mothers, in addition to the above-mentioned macrosomia, are at greater risk of developing many other complications, such as prematurity, periventricular leukomalacia, and birth defects. Moreover, these children more often achieve lower Apgar scores [7,17].

During the pregnancy, there are changes in the lipid metabolism caused by the response to the increased concentration of estrogens and progesterone. The HDL and LDL lipoprotein fractions responsible for the transport of cholesterol cause the accumulation of adipose tissue, and reduced lipolysis increases the risk of obesity [18]. Lipid metabolism at the beginning of pregnancy has an anabolic effect via accumulating energy. Catabolism prevailing in the third trimester generates substrates for the developing fetus. Dyslipidemia disorder during pregnancy causes complications for the woman, the fetus, and also for the newborn [19,20]. Similarly, disturbances in carbohydrate metabolism caused by hyperinsulinism, physiological insulin resistance, and increased levels of cortisol and placental lactogen significantly mobilize adipose tissue [21]. To sum up, pre-pregnancy obesity in women is one of the most important obstetric problems, increasing the risk of both maternal and fetal complications [22].

### 1.2. Characteristics of Selected Adipokines

The adipose tissue serves as an energy store, thermal insulation and protection, and also as an endocrine organ, delivering biologically active substances with hormonal properties called adipokines to the bloodstream.

#### 1.2.1. Leptin

The brain, stomach, breast gland, and placenta release leptin in modest levels 2–3 h after a meal [23]. Blocking neuropeptide Y and suppressing hunger, it regulates appetite. Gluconeogenesis intensifies glucose metabolism, energy utilization, tissue insulin sensitivity and secretion, blood pressure, and angiogenesis [24]. The serum leptin concentration is principally affected by adipose tissue, insulin, glucose, glucocorticosteroids, and TNF-α [25,26]. Leptin is increased in healthy pregnant women’s blood serum [27]. Its serum concentration peaks in the second trimester and lasts until delivery. The placenta produces leptin throughout pregnancy [28].

#### 1.2.2. Adiponectin

Only adipose tissue secretes adiponectin, a protein with anti-inflammatory, anti-atherosclerotic, and anti-diabetic effects. This hormone prevents atherosclerosis, obesity, and insulin resistance by promoting insulin secretion, tissue insulin sensitivity, muscle glucose uptake, inflammation reduction, and lipid reduction. Healthy adults have 5–30 μg/mL of it in their blood serum, but obese people have less [29]. Adiponectin concentration increases in the first trimester due to nutrient storage, then declines by 60% in the second half [30]. Obese pregnant women have decreased adiponectin levels [31]. Umbilical cord blood contains four–seven times more than birthing blood serum [32]. Adiponectin production by the human placenta is contradictory [33,34]. It may regulate placental nutrition transfer to regulate fetal growth [31,35].

#### 1.2.3. Resistin

Resistin increases liver gluconeogenesis to maintain blood glucose [36]. This cytokine inhibits isolated fat cell insulin-stimulated glucose absorption, which may increase hepatic insulin resistance. Low resistin levels may increase insulin sensitivity and de-crease induced hyperglycemia. These findings implicate resistin in diabetes and obesity [37]. Human monocytes and macrophages generate resistin, with adipocytes producing less [38,39,40]. Human plasma resistin ranges from 7 to 22 ng/mL and is favorably linked with fat tissue [29]. The placenta secretes resistin, which may help the developing embryo have enough energy [40]. This may explain its relationship with infant weight [41].

#### 1.2.4. TNF-α

TNF-α’s anticancer and immunomodulatory properties contribute to the development of many illnesses, notably those with an inflammatory background [42,43]. This cytokine was found to regulate body weight in adipose tissue in the early 1990s. Obese people had greater serum TNF-α levels, proportional to their adiposity. Short-term TNF-α exposure inhibited adipose tissue development [44]. However, increased body fat develops TNF-α resistance [45]. TNF-α concentration and receptor affinity may explain the role of this cytokine in obesity pathophysiology. TNF-α is a therapeutic target for inflammatory illnesses such as rheumatoid arthritis, Crohn’s disease, atherosclerosis, sepsis, and obesity [46]. In the third trimester, serum TNF-α concentrations peak [47]. Maternal, fetal, and placental macrophages release TNF-α throughout this time. Many studies have linked pregnancy complications such as preterm fetal bladder rupture, hypertension, and intrauterine growth restriction to increased serum TNF-α levels [47,48].

## 2. Aim of the Study

This study aimed to analyze selected parameters of carbohydrate metabolism, such as: glucose, insulin, insulin resistance index-HOMA-IR and lipid metabolism; total cholesterol, its HDL, LDL, VLDL fractions, triglycerides, as well as adipokines; and leptin, adiponectin, resistin, and TNF-α in maternal and umbilical cord blood sera in pregnancy complicated with preexisting obesity and target BMI. A secondary research goal was to assess the relationship between the assessed adipokines and the BMI of pregnant women and the birth weight of newborns.

## 3. Materials and Methods

### 3.1. Characteristics of the Study and Control Groups

The 61 women in the third trimester of pregnancy hospitalized due to labor at the Department of Obstetrics and Pathology of Pregnancy of the Medical University of Lublin in 2014–2017 gave their informed and written consent to participate in the study. The consent to conduct the research was issued by the Bioethics Committee at the Medical University of Lublin (KE-0254/97/2012). Before starting the study, all patients were interviewed regarding their current health condition, past illnesses, medications, and drug use. The study group (O) consisted of 34 pre-pregnancy obese women whose BMIs, assessed according to WHO criteria, at the beginning of pregnancy were equal to or exceeding 30 kg/m^2^. The control group (C) included 27 pregnant women with lower body weight before pregnancy (BMI 18.5–24.9 kg/m^2^).The study did not include pregnant women diagnosed with factors or comorbidities, such as gestational hypertension, gestational diabetes, connective tissue diseases, autoimmune diseases, or multiple pregnancies.

### 3.2. Methods

The research materials for determining the concentration of the above-mentioned parameters were fasting serum samples separated from the peripheral blood of pregnant women before delivery and from blood collected immediately after delivery from the umbilical vein. About 9 mL of blood was collected in disposable S-Monovette tubes (Sarstedt, Nümbrecht, Germany) containing a blood clotting activator. After 30–40 min, the samples were centrifuged in a centrifuge (Sigma 1–6P, Polygen, Wroclaw, Poland) for 10 min at room temperature. The obtained serum was 200 μL aliquoted in 0.5 mL Eppendorf tubes (Medlab Products, Raszyn, Poland) and stored at −75 °C until the tests were performed. In serum, the concentrations of insulin and glucose, total cholesterol with its fractions (HDL, LDL, VLDL), and triglycerides were determined using standard methods. In the collected material, the concentrations of selected adipokines were also determined using commercial ELISA kits (Enzyme-Linked Immunosorbent Assay): leptin (DRG Instruments GmbH, Marburg, Germany), adiponectin (Mediagnost, Reutlingen, Germany), resistin (Mediagnost, Reutlingen, Germany), and TNF-α (ID Labs™ Biotechnology, Ontario, Canada). In the study and control group, the birth weight of newborns was analyzed and the percentage of macrosomic newborns was assessed. This condition was defined according to the ACOG recommendation as a newborn weight exceeding 4000 g, regardless of the fetal age. For the study and control groups, an analysis of the relationship between selected adipokines and pre-pregnancy BMI of mothers and birth weight of newborns was performed jointly. Based on the concentration of glucose and insulin in the blood serum, the insulin resistance coefficient was calculated according to the following formula: HOMA-IR = (fasting glucose (mmol/L) × fasting insulin (μU/mL))/22.5.

### 3.3. Statistical Analysis

The values of the analyzed parameters measured on the nominal and ordinal scale were characterized by the number and percentage, while those measured on the interval scale by the arithmetic mean, standard deviation, median, 25th, and 75th percentiles, and the range of variation. Due to the skewed distribution of the measured parameters assessed using the W. Shapiro–Wilk test and the heterogeneity of variance assessed using the F-Fischer test, non-parametric tests were used to analyze the existence of differences between the studied subgroups. The Mann–Whitney U-test was used to compare two indep endent groups. The R. Spearman correlation coefficient significance test was used to assess the existence of a relationship between the analyzed measurable parameters. A *p* < 0.05 was assumed as a statistically significant relationship. Statistical analyses were performed based on the Statistica v. 10.0 computer software (StatSoft, Cracow, Poland).

## 4. Results

The clinical characteristics of the study and control groups are presented in Table 1.

The birth weight of newborns in group O was statistically significantly higher than that in group C (*p* < 0.01) (Table 2). The percentage of macrosomic newborns (birth weight > 4000 g) in the O group was statistically significantly higher than that in the C group, 35% (*n* = 12) and 7.4% (*n* = 2), respectively; (*p* < 0.05).

In group O, a statistically significantly higher percentage of caesarean sections was found compared with that of the control group, respectively, 65% (*n* = 22) and 37% (*n* = 10) (*p* < 0.05). No statistical significance was found between the groups in terms of parity and sex of the newborn.

### 4.1. Comparative Analysis of Selected Parameters of Carbohydrate Metabolism in the Study and Control Group

There were no statistically significant differences between group O and group C concerning insulin, glucose, and HOMA-IR concentrations in both maternal and umbilical blood serum. (Table 3)

### 4.2. Comparative Analysis of Selected Parameters of Lipid Metabolism

The concentration of total cholesterol and HDL cholesterol fraction in both maternal blood serum and umbilical cord blood was statistically significantly lower compared with that of the control group (*p* < 0.001). The concentration of TG in the blood serum of the mothers of the studied group was statistically significantly higher compared with that of the control group, the median values (range) were, respectively, 317.5 (123.2–540.4) mg/dL and 280.9 (123.5–376 7) mg/dL, (*p* < 0.05). There were no statistically significant differences between the two groups concerning the concentrations of TG in the umbilical cord blood. There were no statistically significant differences between the study group and the control group about the concentration of LDL cholesterol in the maternal serum. The concentration of LDL cholesterol in the umbilical blood serum in the study group was statistically significantly lower than that in the control group; the median (range) values were 20.6 (1.6–61.2) mg/dL and 31.4, respectively, (19.9–56) mg/dL, (*p* < 0.001). There were no statistically significant differences between the study group and the control group concerning VLDL cholesterol concentrations in maternal blood serum and umbilical cord blood serum (Table 4).

### 4.3. Comparative Analysis of Serum Concentrations of Leptin, Adiponectin, Resistin, and TNF-α in the Study and Control Groups

Leptin levels in both maternal blood serum and umbilical cord blood in the study group were statistically significantly higher than that in the control group. (Table 5).

There were no statistically significant differences between group O and group C concerning adiponectin concentrations in both maternal blood serum and umbilical cord blood. On the other hand, the concentration of resistin in the blood serum of mothers in group O was statistically significantly higher than that in group C and did not differ between groups in the serum of umbilical cord blood.

In terms of TNF-α concentrations in both maternal blood serum and umbilical cord blood, no statistically significant differences were found between group O and group C.

### 4.4. Analysis of the Relationship between the Concentration of Leptin, Adiponectin, Resistin, TNF-α, and Pre-Pregnancy BMI of the Mothers

A positive correlation (r = 0.454) was found between maternal serum leptin concentration and pre-pregnancy BMI (*p* < 0.001).

There was a negative correlation (r = −0.265) between maternal serum adiponectin concentration and pre-pregnancy BMI (*p* < 0.05) 

A positive correlation (r = 0.309) was found between the concentration of resistin in maternal blood serum and pre-pregnancy BMI.

A negative correlation (r = −0.277) was found between the concentration of TNF-α in maternal serum and pre-pregnancy BMI (*p* < 0.05).

There was no correlation between the concentration of leptin, adiponectin, resistin, and TNF-α measured in the umbilical blood serum and the pre-pregnancy BMI of the mothers.

### 4.5. Analysis of the Relationship between the Concentration of Leptin, Adiponectin, Resistin, TNF-α, and the Birth Weight of the Newborn

In the scope of the analyzed adipokines, only a positive correlation (r = 0.379) was found between the concentration of leptin in the umbilical blood serum and the birth weight of the newborn (*p* < 0.001).

## 5. Discussion

Obesity is becoming one of the greatest challenges of the modern world, being a significant health problem for maternal–fetal medicine. Our research aimed to reveal the impact of higher BMI on mothers’ and fetuses’ carbohydrates and lipid metabolism. All studied pregnant women with obesity were screened with 75 g of oral glucose tolerance test on the first obstetrical visit, and only those without any disturbance were enrolled in the study.

In our work, no significant differences were found between the blood glucose concentration in obese and lower-weight pregnant women. This result is similar to the observation of other authors [13,49]. Similar to the results of other studies, no differences in the concentration of glucose in the blood serum of the umbilical cord in obese pregnant women compared to those with lower body weight were found [13,41,49]. When analyzing HOMA-IR in maternal serum, no statistically significant differences were found between obese pregnant women and those with lower body weight. Different results were obtained by Tinius et al. and Catalano et al., presenting a significantly higher HOMA-IR index in obese pregnant women [13,50]. Our studies did not show any differences between the HOMA-IR index in the umbilical cord blood of obese pregnant women compared to those with lower body weight. Other researchers obtained similar results [13,49]. The literature data indicate lower values of HOMA-IR in the serum of umbilical cord blood compared with those obtained in the blood serum of pregnant women, both in the obese and lower body weight categories, which was also confirmed by our study [13,49,50].

In our results, no statistically significant differences were found between the group of pre-pregnant obese women and those of lower weight in relation to serum insulin levels. These results differ from the observations of Tinius et al. and Barker et al., who found higher serum insulin levels in obese pregnant women [13,49]. Our study also did not show a significant difference between obese and lower-weight pregnant women with regard to the concentration of insulin in the umbilical cord blood. The results presented by other researchers are contradictory in this case. For example, Catalano et al. showed an increase in the levels of this parameter in obese mothers, while Barker et al., Tinius et al., and Thakali et al. found no such correlation [13,49,50,51]. It is worth emphasizing, however, that in many of these studies the analyzed groups were heterogeneous, that is, pre-pregnancy obesity coexisted with already existing carbohydrate metabolism disorders, for example insulin-dependent diabetes or gestational diabetes. Moreover, both obese and overweight patients were enrolled in them. This made it difficult to make simple comparisons with the results of our study, which qualified only obese women without the above-mentioned metabolic disorders.

In our research, a significantly lower concentration of total cholesterol in the blood serum of obese pregnant women was reported as compared with lower-weight pregnant women. Similar results were also reported by other authors [24,52,53]. In our study, we also found a significantly lower concentration of total cholesterol in the umbilical blood in the group of obese pregnant women compared with those with lower body weight. This is consistent with the observations of Solis-Paredes et al. [24]. We showed that the concentration of triglycerides in the serum of obese pregnant women was significantly lower compared with the value of this parameter in the serum of pregnant women with lower body weight. These results are consistent with the observations of other authors [24,52,53]. However, in contrast to Solis-Paredes et al., no significantly lower concentration of triglycerides in the umbilical cord blood of obese pregnant women compared to those of lower body weight was found [24]. Analyzing the concentration of HDL cholesterol, lower concentrations of this parameter in the blood serum were found in obese pregnant women compared with those of lower weight, similarly to other authors [24,53]. This relationship also concerned the levels of this parameter in the serum from umbilical cord blood. Contrary to Bozkurt et al., Meyer et al., and Solis-Paredes et al., we did not find significant differences between the group of obese pregnant and non-obese pregnant women concerning serum LDL cholesterol levels. The cited authors observed statistically lower values of this parameter in the first of these groups [24,52,53]. Only a few studies have investigated the adipokines in mother–newborn pairs during pregnancy complicated by obesity and healthy ones and reported about interactions with clinical obstetric variables or other metabolic parameters. Furthermore, a comparison of existing studies is difficult due to differences between assays, sample collection, gestational age, and patient characteristics.

In our research, statistically significantly higher serum leptin levels were found in obese pregnant women compared with those with lower body weight. These correlations were observable both in the blood serum of the subjects and in the umbilical cord blood, which is consistent with the other authors’ reports [54,55,56]. As already mentioned, leptin acts as a growth factor for the fetus, depending on the nutritional status of the mother. In our study, we did not point any correlation between the concentration of leptin in the blood serum of pregnant women and the birth weight of their offspring. This is consistent other literature data; a few of them described such a relationship [54,57,58,59]. In our study, the concentration of leptin in the umbilical cord blood of obese pregnant women was significantly higher than that in those with lower body weight. Additionally, we observed a positive correlation between the values of this parameter in umbilical cord blood and the birth weight of the newborn, which is consistent with the reports of other authors [60,61,62].

Another adipokine that plays a role in the pathomechanism of insulin resistance (IR) is adiponectin. Indeed, an inverse relationship has been demonstrated between its plasma concentrations and IR [63]. It has been suggested that adiponectin reduces hepatic glucose production, enhances insulin action in this organ, and stimulates the supply of glucose in skeletal muscles [64,65]. Contrary to other adipokines, such as the previously discussed leptin and TNF-α, the level of adiponectin in the blood serum is lower in obese people, including non-pregnant women, compared with that in people with lower body weight [29,66]. In our research, however, no such differences were found between obese pregnant women and those with lower body weight. This is consistent with most of the literature data [32,48,55]. However, Zembala-Szczerba et al. found a significantly lower concentration of adiponectin in the blood serum of obese pregnant women compared with those with target BMI. It should be noted that they also included patients with GDM and hypertension, which could have influenced the results [67]. Nevertheless, in our study, a negative correlation was found between the concentration of adiponectin in the blood serum of pregnant women and their pre-pregnancy BMI. Vernini et al. also noted a negative correlation between the concentration of adiponectin and the gestational BMI, suggesting that the greater the obesity, the greater the insulin resistance and the lower the adiponectin [68]. This result is different from that obtained by Wang et al. [69]. Similar to other authors, we found no correlation between maternal adiponectin concentration and the birth weight of the newborn [55]. In turn, according to Lowe et al., the level of this adipokine in the blood serum of healthy pregnant women with lower body weight negatively correlates with the birth weight of their children [70]. However, taking into account the role of adiponectin in glucose metabolism and tissue insulin sensitivity and its participation in the transplacental transport of insulin-dependent amino acids, it seems that it may be a regulator of fetal body weight [32,71,72]. For example, it has been shown that its higher concentrations in umbilical cord blood are associated with a higher birth weight of the newborn [53]. Although the high molecular weight of adiponectin should prevent it from penetrating the placental barrier, the reported differences in its concentrations between maternal serum and umbilical cord blood suggest the existence of specific transport mechanisms [69]. Chen et al. believe that it occurs with the participation of other cytokines, such as TNF-α, interferon-α, IL-6, and leptin [73]. In our study, no significant differences were found in umbilical cord blood adiponectin levels between obese pregnant women and those with lower body weight. Similar results were obtained by Lindsay et al. [71]. However, they differ from the observations of Brynhildsen et al., who found significantly higher concentrations of this adipokine in the umbilical cord blood of obese pregnant women, which were also two or three times higher compared with the levels observed in the blood serum of healthy adults [55]. Additionally, Wang et al. observed a negative correlation between the umbilical adiponectin concentration and the mother’s BMI, which was not confirmed in our study [69]. The results presented in the literature may indicate the role of adiponectin in fetal growth and its potential influence on the occurrence of neonatal macrosomia [74,75].

The results of research on resistin in obese patients are inconclusive. Some of them indicate higher concentrations of this adipokine in the blood serum of patients with obesity compared with people with a target BMI, while others prove that it is quite the opposite [59,62,63,64,65,66,67,68]. This may be due to imperfect methodology with the laboratory tests used, as well as the possibility of cross-reactions with resistin-like particles circulating in the blood serum [24]. The concentration of resistin increases with the development of pregnancy; a positive correlation of its concentration with gestational age was observed with the subsequent decrease in its concentration after delivery [69]. In our study, statistically significantly higher concentrations of resistin in the serum of obese pregnant women were found compared with those with lower body weight. These results differ from those obtained by Nien et al., who showed no difference. However, not only pregnant, obese, but also overweight ones were qualified for their research [76]. When analyzing the concentration of resistin in the umbilical cord blood, no significant differences were found between the group of obese pregnant women and those of lower weight. Similar results were presented by Wang et al. [69].

The studies conducted by Kirwan et al. presented that the concentration of TNF-α in the serum of pregnant women was the most important independent predictor of insulin sensitivity, and the higher concentrations achieved in the third trimester of pregnancy inversely correlated with insulin resistance [77]. In in vitro studies, the cited authors showed that most, as much as 94%, of placental TNF-α is released into the mother’s circulation, and only 6% is released into the fetal circulation. It has been proven that high levels of this parameter in the blood serum inhibit the secretion of adiponectin from fat cells and lower its concentration in the serum, which in turn increases insulin resistance [77]. In our study, no statistically significant differences were found between the serum TNF-α concentration in obese pregnant women compared to those with lower body weight before pregnancy. Similar results were presented by other researchers [67,78]. In our study, no statistically significant difference was found in the concentration of TNF-α in the serum of umbilical cord blood between the group of obese and lower-weight pregnant women. Conflicting results were obtained by Challier et al., where the compared groups of pregnant women were heterogeneous, including both obese and overweight pregnant women, while the control group consisted of women with both lower and deficient body weight [79]. We found a negative correlation between the concentration of TNF-α in the serum of pregnant women and BMI before pregnancy. On the other hand, Aye et al. obtained opposite results [80]. In our study, no correlation was found between TNF-α in the serum from umbilical cord blood and the birth weight of the newborn. The presented reports on this subject are inconsistent. Some, such as Aye et al., obtained similar results, while others, such as de Toledo Baldi et al., presented a positive correlation between the above mentioned parameters [79,80]. Additionally, the study by Aye et al. is coherent with the observations of other authors suggesting that the inflammation associated with maternal obesity affects the developing fetus not directly by increasing the concentration of proinflammatory cytokines in the umbilical blood serum, but possibly by altering the functioning of the placenta [79]. Long-term obesity may lead to the development of tissue resistance to TNF-α, which contributes to the development of insulin resistance [81]. Experimental studies from the 1990s showed that the administration of TNF-α led to a decrease in insulin sensitivity, and the use of anti-TNF-α antibodies led to a reversal of this phenomenon [82]. Additionally, the influence of TNF-α on the increase in serum leptin concentration was demonstrated, probably at the post-translational stage [83].

The clinical Implication of this study is a need to open a discussion on the policies and recommendations for pre-pregnant obese women undergoing medical treatment for, for example, antenatal corticosteroids therapy (ACS). Our team started a longitudinal, prospective observational/experimental study on the benefits of daily glucose monitoring in high risk (obese) pregnant patients during ACS. This study was approved by the Bioethical Commission at the Medical University of Lublin (Res No. KE-0254/98/04/2022) [84].

One of the limitations of our research is the performance of analyses in obese patients by calculating their BMI. The BMI index itself is an imperfect indicator of adiposity. The measurement of body composition, including body fat percentage, might more accurately identify the obese woman at risk [85,86].

## 6. Conclusions

It does not seem that obesity significantly affects the basic parameters of the carbohydrate metabolism of a pregnant woman, in particular the level of glucose, serum insulin, and the insulin resistance index (HOMA-IR). Obesity disturbs the lipid profile of a pregnant woman, which is manifested by a significant increase in the concentration of triglycerides and a decrease in the level of HDL cholesterol in the serum. Obesity increases the concentration of leptin and resistin in the serum of pregnant women, which hypothetically may be caused by the increased volume of adipose tissue. The concentrations of leptin and resistin in the blood of pregnant women correlate positively, and the concentrations of adiponectin and TNF-α negatively correlate with their pre-pregnancy BMI values. There is a positive correlation between the concentration of leptin in the serum of umbilical cord blood and the birth weight of the newborn, which suggests that this parameter contributes to the pathomechanism of macrosomia.

## Figures and Tables

**Table 1:** Clinical characteristics of the study group (O) and control group (C).

General Characteristics		Median	Percentile		Range
			25	75	
Age (years)	O	30.5	28	33	25–43
	C	27	25	32	21–38
Height (cm)	O	167	160	171	150–179
	C	165	162	170	155–184
Weight before pregnancy (kg)	O	94	79	103	69–130
	C	59	54	63	45–73
BMI before pregnancy (kg/m^2^)	O	32.7	30.1	35.6	30–44.5
	C	21.2	19.4	22.8	18.5–25
Body weight in third trimester (kg)	O	108.5	95	115	85–138
	C	73	67	78	59.5–100
Systolic blood pressure (mmHg)	O	131	117	131	109–136
	C	118	105	128	110–134
Diastolic blood pressure (mmHg)	O	83	71	83	68–88
	C	79	69	81	67–86
Weight gain during pregnancy (kg)	O	12	9	16	3–28
	C	14.75	11.5	19	8–30
Gestational age (weeks)	O	40	39	40	37–41
	C	40	39	40	37–42

**Table 2:** Birth weight of newborns in the study (O) and control (C) groups.

Neonatal Birth Weight (g)						
		Median	Percentile		Range	Statistical Analysis
			25	75		
Neonates	O	3745	3490	4170	2880–4420	*p* < 0.01
	C	3310	3100	3630	2940–4500	

Statistically significant results (*p* < 0.05) highlighted in italic. Values are median and interquartile range (25th–75th percentiles). *p*-values from unpaired *t*-test for continuous parametric variables.

**Table 3:** Maternal (M) and cord blood (U) insulin, glucose concentration, and HOMA-IR in the study group (O) and control group (C).

		Median	Percentile		Min–Max	Statistical Analysis
			25	75		
Insulin (M) (µlU/mL)	O	14.0	10.4	20.4	3.2–55.6	*p* = 0.7
	C	12.9	9.2	21.4	4.4–68.4	
Insulin (U) (µlU/mL)	O	6.7	4.5	11.2	2.2–30	*p* = 0.88
	C	8.1	4.5	9.4	2.8–14.1	
Glucose (M) (mmol/L)	O	4.7	3.6	5.4	1.9–6.7	*p* = 0.95
	C	4.3	3.9	5.2	2–6.6	
Glucose (U) (mmol/L)	O	3.8	3.0	4.7	0.8–7.3	*p* = 0.59
	C	3.7	3.1	5.7	0.6–6.4	
HOMA-IR (M)	O	2.4	1.6	4.4	0.6–15.3	*p* = 0.91
	C	2.5	1.5	4.5	0.7–19.1	
HOMA-IR (U)	O	1.1	0.7	2.0	0.2–3.7	*p* = 0.86
	C	1.3	0.8	1.8	0.3–3.7	

Values are median and interquartile range (25th–75th percentiles). *p*-values from unpaired *t*-test for continuous parametric variables and Mann–Whitney U-test for nonparametric variables.

**Table 4:** Concentrations of selected parameters of lipid metabolism in the serum of mothers’ blood (M) and umbilical cord blood (U) in the study (O) and control (C) groups.

Substance		Median	Percentile		Range	Statistical Analysis
			25	75		
Total cholesterol M (mg/dL)	O	265.5	242.4	288	205.1–385.1	*p* < 0.05
	C	296.5	259	319.9	185–398	
Total cholesterol U (mg/dL)	O	53.7	42	70.2	32.7–105.9	*p* < 0.001
	C	78	67.6	87	53.2–110.6	
TG M (mg/dL)	O	317.5	267.9	350.9	123.2–540.4	*p* < 0.05
	C	280.9	203.0	303	123.5–376.7	
TG U (mg/dL)	O	26.1	13.8	43.7	1–73.4	*p* = 0.67
	C	28.9	19.1	39.9	13.7–80.4	
HDL M (mg/dL)	O	61.2	53.2	68.8	36.7–102.9	*p* < 0.001
	C	76.8	66.1	83	42.7–115	
HDL U (mg/dL)	O	18.2	11.3	30.7	9.4–41.2	*p* < 0.001
	C	35.6	34.1	40.2	29.8–49.7	
LDL M (mg/dL)	O	162	138.6	190.7	98.7–267.7	*p* = 0.99
	C	168.8	147	188.5	95–216.2	
LDL U (mg/dL)	O	20.6	14.6	30.2	1.6–61.2	*p* < 0.001
	C	31.4	26.8	39.1	19.9–56	
VLDL M (nmol/mL)	O	546.5	498	1418.7	320.3–7422.6	*p* = 0.24
	C	666.7	555.7	1085.8	415.5–7748.3	
VLDL U (nmol/mL)	O	576	506.1	1566.6	360.5–7107.2	*p* = 0.48
	C	697.9	522.1	1690.1	402.1–7050.4	

TG—triglycerides, HDL—high density lipoproteins, LDL—low density lipoproteins, VLDL—very low density lipoproteins. Statistically significant results (*p* < 0.05) highlighted in italic. Values are median and interquartile range (25th–75th percentiles). *p*-values from unpaired *t*-test for continuous parametric variables and Mann–Whitney U-test for nonparametric variables.

**Table 5:** Concentrations of selected adipokines in the serum of maternal blood (M) and umbilical cord blood (U) in the study group (O) and control group (C).

Adipokines		Median	Percentile		Range	Statistical Analysis
			25	75		
Leptin M (ng/mL)	O	36.4	27.9	54.1	4.1–124.4	***p < 0.001***
	C	16.5	10.1	34.3	4.2–95	
Leptin U (ng/mL)	O	10.7	6.8	22.0	1.7–51.3	***p < 0.05***
	C	7.0	4.4	13.1	1.1–20.1	
Adiponectin M (μg/mL)	O	5.9	4.4	8.5	2.5–18.4	*p* = 0.08
	C	8.4	5.1	10.4	2–18	
Adiponectin U (μg/mL)	O	29.0	24.2	34.9	15.4–41.8	*p* = 0.4
	C	33.4	24.0	37.1	10.9–4.0	
Resistin M (ng/mL)	O	13.4	9.1	17.8	5.4–30.4	***p < 0.05***
	C	8.9	7.4	12.7	4.9–24.7	
Resistin U (ng/mL)	O	14.4	12.0	20.6	8.9–56.8	*p* = 0.24
	C	12.6	11.4	18.6	8.2–22.7	
TNF-α M (pg/mL)	O	10.1	3.1	32.7	0.001–2001.9	*p* = 0.13
	C	26.2	6.1	257.2	0.006–1906.5	
TNF-α U (pg/mL)	O	24.8	4.6	50.4	0.006–1526.3	*p* = 0.8
	C	12.3	6.1	50.2	0.4–972.2	

TNF-α—tumor necrosis factor alpha. Statistically significant results (*p* < 0.05) highlighted in italic. Values are median and interquartile range (25th–75th percentiles). *p*-values from unpaired *t*-test for continuous parametric variables and Mann–Whitney U-test for nonparametric variables.

## Data Availability

Data supporting the findings of this study are available from the corresponding author [A.S] on reasonable request.

## Abbreviations

BMI	Body Mass Index
HOMA-IR	Homeostatic Model Assessment Insulin Resistance
VLDL	Very Low Density Lipoproteins
TG	Triglycerides
HDL	High Density Lipoproteins
LDL	Low Density Lipoproteins
TNF-α	Tumor Necrosis Factor alpha
IR	Insulin Resistance
